# Hernia of the Dental Pulp

**Published:** 1874-05

**Authors:** E. M. Morrison

**Affiliations:** Des Moines, Iowa


### HERNIA OF THE DENTAL PULP.

BY E. M. MORRISON, M.D., DES MOINES, IOWA.

A hernia according to Dunglison is: "Any tumor formed by the displacement of a viscus or a portion of a viscus, which has escaped from its natural cavity by some aperture and projects externally."

The dental pulps are not classed with the viscera, but like them are encased or enclosed in central cavities. The bony walls of the teeth protect them even more secure than the viscera are protected. The brain itself is not so well protected, but, for all this, the pulps are subject to abnormal conditions, if not identical with, at least similar to the hernia of these organs, and we can see no great impropriety or violence to our nosonomy in extending the term to include them also.

To get at the points we wish to notice, we will commence with exposed pulps. It would be interesting, however, to discuss the various causes leading to exposure of the pulp, and the phenomena attending this morbid condition, but we have here a sufficiently well defined starting point for our present purposes.

When the pulp of a tooth becomes exposed from any cause, it is subject to a series of pathological conditions and accidents; generally precursors of inflammation, though this condition is not always developed. It is stated in works on dentistry that exposure of the pulp is a cause of odontalgia, but this is not strictly correct: for many pulps are exposed for months, and perhaps years, without the development of this phenomenon. In fact, they even die without pain, or other of the usual preliminary symptoms of inflammation. However, if the pulp or a portion of it with its lining membrane protrude through a constricted aperture of exposure, in such a manner as to become strangulated, pain of the most excruciating character will be the result. This is the only form of toothache worthy of the name—odontalgia, but it may be followed by odontitis, periostitis and alveolar abscess, all of which are known by the generic name—toothache in common parlance.

The pain results from pressure upon the nervous filaments caught or impacted in the constricted aperture, and is similar to that of a nerve accidentally ligated in a surgical operation or strangulated hernia in general, only modified by the character of the tissues or organs involved. The blood, or liquor sanguinis at least, is forced through the aperture of exposure by the systaltic action of the heart, and the diastole can afford no means for its return; a hernia tumor is formed and there is no relief till the pathological conditions are changed by natural processes, remedial agents or the death of the part.

Hernia of the pulp is of frequent occurrence, but not always strangulated.

Harris speaks of "fungus of the pulp," which is only a variety of hernia not thus incarcerated.

The numerous failures in capping have been mainly due to the occurrence of this affection, but the true rationale was not well understood or attributed to this cause. The space between the aperture of exposure and the cap was generally soon occupied by the protruding pulp, and this abnormal condition could not be sustained long, except in a very few instances. Pain and the other usual preliminary symptoms of inflammation were looked for in vain, in many cases, and their absence was well calculated to mislead, but, under the circumstances, they were necessarily wanting or could not be detected.

The present methods of operating are more successful, mainly because there is no space left above the aperture of exposure for the protrusion of the pulp, and there can be no hernia. A portion of the success however is due to a general advancement in therapeutical knowledge, and the substitution of a better and more genial class of non-conductors.

Inflammation, according to Dr. Bennett,* is an exudation of the liquor sanguinis. "Indeed," says this author, "it is only

*Bennett's practice of Med. p. 162.

when the exudation has occurred that we can ever feel satisfied that even the tendency existed. It follows that no one of the preliminary phenomena, or all of them combined, constitute an inflammation unless exudation has occurred; so that, for all practical as well as scientific purposes, it may be said that this morbid state consists essentially of an exudation of liquor sanguinis."

We shall not stop to discuss the theories or phenomena of inflammation in detail, yet it will be proper to state that the symptoms attending and preceding it are not constant. Sometimes there are pain, heat, redness and swelling, but these conditions are seldom all present, and in some cases reported, not one of them could be detected during the life of the individual, yet in a cadaveric autopsy, the results of inflammation were found so distinct and well defined as to preclude the possibility of a doubt, as to its having existed. In dental practice, however, we seldom fail to find the usual precursros of them, but dead pulps, alveolar abscesses and other evidences are sometimes found where none of these systoms had been observed by the patient or the dentist. Again, we may find these morbid preliminary phenomena, and no exudation or inflammation proper will be the result. In fact, a threatened inflammation is not unfrequently averted, by the recuperative powers of the system, or by proper treatment.

In dental hernia we should observe these facts, and base our applications and treatment upon sound surgical and therapeutical principles bearing in mind that the pulp is a delicate, highly sensitive organ, responsive to the action of medicines, and susceptible of cure. We have simply to treat the pathological conditions which precede or accompany inflammation. Our treatment should be instituted as early as possible ; for the hope of saving the pulp alive will diminish as time lapses, and the symptoms indicate a fully developed inflammation.

In the strangulated variety nature, if left alone, goes vigorously to work to relieve this painful condition, by a kind of gangrenous exfoliation, and our efforts should be to aid and hasten the operation, so as to cut short the pain and gangrenous process. As the pulp is not a viscus, we may here deviate from the usual practices of surgery in visceral hernia, where it is necessary to save the entire organ, to maintain the life of the individual. The loss of a portion of the pulp is not imcompatible with life, and we may remove the hernial protrusion without seriously endangering even the life of the organ. To accomplish this successfully, all foreign substances should be removed from the cavity of exposure, so that the pulp may be well uncovered ; then an application of carbolic acid should be made directly to it, and covered over with sandarac varnish, or other proper substance to protect the mucous membranes of the mouth from its effects. This treatment will generally afford relief almost immediately. But thus destroying the protruding portion of the pulp, or so much of it as will permit its recession into its proper chamber, we accelerate the accomplishment of the work that nature intended. The same result may be accomplished by excision, or by any active cautery or escharotic that will not too greatly endanger the life of the pulp. This treatment, of course, is based upon the presumption that the general health is good, and that all the circumstances are favorable, so we need not here enumerate all the adjunctives probable, and sometimes required, where there is complication.

In the variety of hernia described by Dr. Harris as fungus of the pulp, we have not had much experience, but owing to the greater care of the teeth, or other causes, it is perhaps not so frequently met with now as formerly. We belive Dr. Harris treatment is too radical for the present status of the profession, and that we should be more conservative. He says : "The remedial indication in such cases consists in the removal of the tooth. A cure can not be effected by extirpating the morbid growth. The author has frequently removed them nearly to the extremity of the root, but they have always reappeared in a few days or weeks after the operation. But even if a return of the disease could be prevented, still, the extraction of the tooth should be insisted upon, as all teeth in which tumors of this sort are situated, have become morbid irritants."

This affection, however, is deemed curable in some cases, and under favorable circumstances, we would excise and apply carbolic acid with a compress over the aperture of exposure to prevent the recurrence of the morbid growth. If there should be great sensitiveness, as is sometimes the case, we would apply chloroform and carbolic acid to the tumor to obtund the pain before the operation. In cases of this kind, where the pulp is not too much diseased by inflammation, we can see no barrier to success that would seem to defy all treatment, and we believe that judicious medication and manipulation will be rewarded with success. But, if the life of the pulp can not be saved, it will not neccessarily follow, even then, that the tooth must be sacrificed in every case. There will be plenty of room yet left for the exercise of judgment and conservative skill, but to enumerate all the conditions and indications that suggest themselves here would be a waste of room and time.

As soon as the pulp is restored to health, if restored at all, it is neccessary to fill the cavity in a substantial manner, being careful to close the aperture of exposure securely with a good non-conductor, as is essential in all cases of exposed pulps. The test for restored health is the same as in other cases of exposed pulps ; by a temporary or test filling of Hill's stopping or other material that can be easily removed if necessary for further treament.

We have had patients with dental hernia and aching teeth, to apply for their extraction, but the teeth were treated in the manner described, and their pulps saved alive and restored to health. Some of these cases have been under the test of active service for more than ten years, and have manifested no morbid conditions to disturb their possessors.

